# This abstract has also been published as O42

**DOI:** 10.1186/1742-4690-8-S2-P64

**Published:** 2011-10-03

## This abstract has also been published as O42

This abstract has also been published as O42

